# Short-Term Clinical Assessment of Treating Female Androgenetic Alopecia with Autologous Stem Cells Derived from Human Hair Follicles

**DOI:** 10.3390/biomedicines12010153

**Published:** 2024-01-11

**Authors:** Katarzyna Krefft-Trzciniecka, Zuzanna Piętowska, Alicja Pakiet, Danuta Nowicka, Jacek C. Szepietowski

**Affiliations:** Department of Dermatology, Venereology and Allergology, Wrocław Medical University, 50-368 Wrocław, Poland

**Keywords:** androgenetic alopecia, stem cell therapy, hair follicle stem cells, autologous cell micrografts, regenerative medicine

## Abstract

Background: Androgenetic alopecia (AGA) is the most common form of alopecia, but treatment options are limited. This study evaluated clinical improvement in hair condition in women with AGA six months after a single injection of autologous cell micrografts (ACMs) containing hair follicle stem cells and dermal papilla cells. Methods: Twenty-three women with clinically and dermoscopy-confirmed AGA were included. Five 2.5 mm punch biopsies were taken from the skin of each patient with the Regenera device. The cell suspension was prepared with the Rigeneracons device and then injected into the hormone-dependent hairy zone of the scalp. Results: A significant improvement was observed on the visual analog scale (VAS) when comparing pre- and post-procedure photos (*p* < 0.001). The change in VAS scores was moderately negatively correlated with baseline ferritin concentration and positively with iron concentration. Improved outcomes were associated with higher baseline levels of sex hormone-binding globulin and 17α-hydroxyprogesterone. Neither testosterone nor DHT showed a significant correlation with VAS scores. Conclusions: The ACM procedure was shown to be both safe and effective, yielding satisfying results six months after a single treatment session. Future investigations should aim to gather evidence that enables the development of a cost-effective approach while minimizing treatment burden and costs.

## 1. Introduction

Androgenetic alopecia (AGA) stands as the most common form of alopecia globally [1]. Both male and female types of hair loss are characterized by a gradual conversion of terminal hair into miniaturized hair. AGA affects at least 80% of men and 50% of women by the age of 70, with a higher incidence among Caucasians [2]. The inheritance patterns of AGA are polygenic, involving complex inheritance from either or both sides of the family [3]. In individuals genetically predisposed, androgens play a causal role in AGA. In individuals with AGA, there is an increase in 5-reductase activity and elevated dihydrotestosterone (DHT) levels in hair follicles for both women and men. Testosterone undergoes conversion by 5-alpha-reductase to DHT, which is considered the underlying cause of hair follicle miniaturization [4,5,6,7].

During hair follicle morphogenesis and regular hair growth, hair follicle stem cells (HFSC) and dermal papilla cells (DPCs) play a leading role [8]. In AGA, the differentiation of HFSCs is hampered by factors secreted by dermal cells [9]. It is assumed that HFSCs are located in the bulge region of the outer root sheath of the hair follicle [10]. DPCs, originating from the hair follicle, form a superficial papilla located at the base of the hair follicle, surrounded by a large number of androgenic receptors [11]. HFSCs and DPCs ensure conducive conditions for hair regeneration. In scarring alopecia, occurring, for example, in planus lichen and lupus erythematous, provocative cell invasions around the bulge lead to irreversible loss of HFSCs. Despite damage to the progenitor cells, HFSCs remain preserved in AGA, making this type of hair loss reversible [12].

Society continually seeks effective interventions to reduce the incidence and economic burden of mental and physical illnesses associated with alopecia. Consequently, therapies and treatment products for alopecia have been widely studied for decades [13]. Currently, only two drugs for AGA, topical minoxidil and oral finasteride, have received approval from the US Food and Drug Administration (FDA). Finasteride competitively inhibits types II and III 5-alpha-reductase isoenzymes, preventing the conversion of testosterone to dihydrotestosterone. While it slows hair loss in the treatment of AGA, it does not halt the process completely. Given potential adverse effects such as decreased libido, erectile dysfunction, reduced ejaculatory volume, and gynecomastia, alternative methods are often favored by men experiencing AGA [14]. The formulation of topical minoxidil to treat AGA was developed in 1987. It is used both for men and women to stimulate hair growth; however, the mechanism of action remains poorly understood. Clinically, minoxidil shortens telogen and stimulates hair follicles to enter anagen, the duration of which it also extends. The effect of this substance is to increase hair thickness and length. The sulfotransferase enzyme in the human scalp transforms minoxidil into its active form, minoxidil sulfate, but variations in sulfotransferase activity between individuals impact the efficacy of minoxidil [15]. In addition to approved therapies, many non-FDA-approved treatments have demonstrated effectiveness in treating AGA [16]. Regenerative medical therapies, such as stem cells and platelet-rich plasma therapies, are currently reported as promising interventions for alopecia [17,18]. The molecular mechanisms that underlie the effects of these regenerative therapies remain elusive. Stem cells possess essential properties, including self-renewal, migration, anti-inflammatory, and immune modulation properties, which are crucial for tissue and organ repair. Initially, it was believed that the therapeutic effects of SCs were based on their ability to migrate to damaged tissue and then differentiate to replace damaged tissue or organs. However, Gnecchi et al. found that the therapeutic effects of stem cells on diseased tissues were, at least partially, caused by the release of trophic factors (paracrine) [19,20]. In our recent systematic review, we analyzed the use of different stem cells for the treatment of AGA, encompassing 15 studies with 653 female and male patients. This study illustrates the effects of different stem cells on hair density. The main conclusion drawn from the analysis was that stem cell therapy has a positive effect on hair density regardless of the hair origin [21].

Autologous cell micrograft (ACM) is a method used to extract autologous mature stem cells from the patient’s scalp biopsy through preparatory systems for mechanical disintegration and solid tissue filtering. Gentile et al. [22] examined 27 samples of the micrografts to identify HFSCs. Their calculations showed that each micrograft contained about 4000 cells, of which DPCs constituted about 4.6%, while HFSCs comprised 2.4%. The proposed mechanism of action of ACMs in AGA is to enhance the regeneration of hair cells by transplanting mature multipotent stem cells and to restore hair growth signals by injecting growth factors. However, the exact mechanism has not yet been established [23]. In this article, we describe the short-term clinical efficacy of a single application of ACM, obtained with the Regenera Activa^®^ device to treat female AGA. The aim of this work was to evaluate clinical improvement in hair condition, specifically hair pigmentation and density, obtained through a single injection of ACMs containing HFSCs and DPCs.

## 2. Materials and Methods

### 2.1. Study Overview

The primary objective of the study was to compare clinical effects observed in images taken before and six months after a single session of ACM. Regenera Activa^®^ (Human Brain Wave SRL, Turin, Italy) is a technology that uses the Regenera Activa^®^ device, which is a preparative system for mechanical disintegration and filtering of solid tissues to extract stem cells [24]. The procedure is conducted using the Rigenera HBW method (Regenera^®^ Protocol, Rigenera^®^ Activa, Human Brain Wave SRL, Turin, Italy), developed in Italy in 2013 and available in over 50 countries. In this method, ACM is prepared in a 3-step protocol. Initially, the treatment involves mechanically disintegrating a tissue sample obtained through a skin punch. Subsequently, the sample is filtered (50 microns) and administered intradermally to the affected area following technical specifications. This technique has been found to be beneficial in dermatology, esthetic medicine, wound care, orthopedics, and rehabilitation, as well as oral surgery and dentistry for regenerating various tissues. The protocol involves using several devices: Regenera Activa^®^ to collect punch biopsies, Rigeneracons (Human Brain Wave SRL, Turin, Italy) to produce a cell suspension used as an ACM, and a standard syringe to inject an ACM into target areas. All devices are CE-certified as Class I medical devices [25]. The protocol for regenerating tissues is described in various publications [26,27,28,29].

Four dermatology specialists used the visual analog scale (VAS) [30] for the assessment and presentation of treatment effects. The diagnosis of AGA was established through a comprehensive evaluation, including detailed treatment history, clinical tests, blood tests, and trichoscopy. The severity of AGA was assessed based on the Ludwig scale [31].

### 2.2. Patients

The study included 23 patients who were clinically and dermoscopy-confirmed to have been diagnosed with AGA in grades 1–3 according to the Ludwig scale. The main dermatoscopic features of AGA were hair shaft thickness heterogeneity, yellow dots, and perifollicular hyperpigmentation [32]. Primary exclusion criteria included immunosuppression, cancer, severe chronic diseases, pregnancy, breastfeeding, age under 18 years, hormonal contraception, hyperprolactinemia, hypothyroidism, positive ANA3 antibodies, active inflammation of the scalp, coagulation disorders, lignocaine allergy, and unstable emotional state. Patients with positive ANA3 were also excluded. ANA is an antibody class that binds to cell components of a cell nucleus. These proteins are usually divided into two groups: antibodies against DNA and histones and antibodies against nuclear material. Of the entire population, 20–30% have ANA antibodies. The presence of ANAs and their subtypes increases the likelihood of systemic autoimmune diseases but does not necessarily confirm the onset of autoimmune diseases [33,34,35]. Patients who had received oral treatments (finasteride, dutasteride, minoxidil, antiandrogens) and topical treatments (minoxidil, prostaglandin analogs, corticosteroids) for AGA in the past six months were excluded. Additionally, patients who used medical devices such as low-level laser therapy or underwent procedures such as platelet-rich plasma or micro-needling were not included in the study. Prior to the enrollment, all patients gave written, informed consent for participation in the study. This study was conducted in compliance with the Declaration of Helsinki of the World Medical Association. The study protocol was approved by the local ethics committee (approval no. 1074/2021).

Biochemical parameters were analyzed as follows: electrochemiluminescence assays for thyroid-stimulating hormone (TSH), anti-thyroid peroxidase antibodies (anty-TPO), antithyroglobulin antibodies (anti-TG), testosterone, sex hormone-binding globulin (SHGB), prolactin, and cortisol; and colorimetric assays for iron were analyzed with cobas^®^ e 411 (Roche Diagnostics GmbH, Mannheim, Germany). Chemiluminescence assays for androstenedione, vitamin D3, folic acid, ferritin, and vitamin B12 were performed on LIAISON^®^ XL (DiaSorin, Saluggia, Italy). Diagnostic laboratory kits from EUROIMMUN, Wroclaw, Poland) kits were used for enzyme-linked immunosorbent assays for dehydroepiandrosterone sulfate (DHEA-S), dihydrotestosterone (DHT), 17α-hydroxyprogesterone, adrenocorticotropin (ACTH), and for immunoblotting of antinuclear antibodies (ANA), which were analyzed on EUROBlot One (EUROIMMUN, Wroclaw, Poland). Hematology was performed on Sysmex XN-1000 (Sysmex, Norderstedt, Germany). For every test, the kits and reagents were purchased from the instrument manufacturer and operated according to the instructions provided.

### 2.3. ACM Procedure

**Figure 1:**
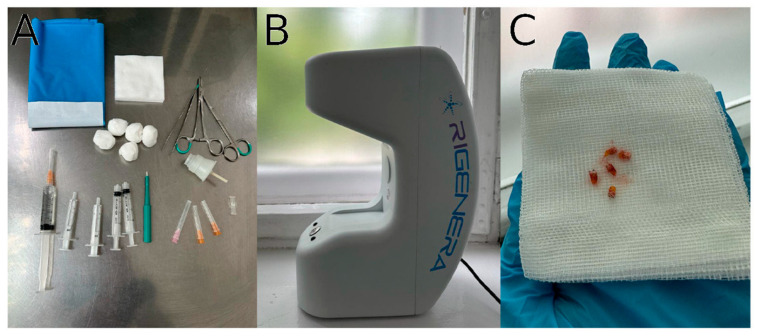
Autologous cell micrograft treatment protocol elements. Necessary tool kit (**A**); Rigenera^®^ class I medical device (**B**); five micrograft biopsies from the skin behind the ear (**C**).

Under local anesthesia, five punch biopsies with a diameter of 2.5 mm each were taken from the skin behind the patient’s ear. The collected micrografts and the necessary equipment are shown in Figure 1. Subsequently, the collected samples were placed in the Rigeneracons and covered with 2 mL of sterile physiological solution. The cell suspension was then generated by rotating Rigeneracons at 80 RPM for 2 min. Following this, the obtained suspension was diluted with an additional 2 mL of sterile physiological solution. The resulting solution was injected subdermally into the scalp hair area using a 1 mL syringe and a 30 mm needle. Each injection point received 0.1 mL of the solution, spaced at about 1 cm between needle punctures. Importantly, only the hormone-dependent hairy zone of the scalp was subjected to the therapy.

### 2.4. Clinical Evaluation of Hair Growth

To evaluate the effects of the treatment, pictures of the patient’s scalps were taken before and six months after the treatment session. The pre- and post-treatment images were taken in the same room, under similar lighting conditions, and with patients in the same head position. Four dermatology specialists independently evaluated the pre- and post-treatment images using the VAS scale. Additionally, following the treatment, the principal researcher recorded the patients on the Ludwig scale.

### 2.5. Statistical Analysis

The data analyses were performed using SigmaPlot version 14.5 (Systat, Software Inc., San Jose, CA, USA). The distribution of data was verified using the Shapiro–Wilk test. Variables with normal distributions were presented as mean ± standard deviation (SD) and those without normal distribution were presented as medians with interquartile range (IQR). To investigate differences before and after treatment, paired *t*-tests or Wilcoxon signed-rank tests were utilized according to the distribution of the variables. Associations between baseline clinical characteristics and clinical outcomes were further evaluated by calculating Spearman correlation coefficients.

## 3. Results

### 3.1. Baseline Characteristics of Study Patients

A total of 23 patients were female, with a mean (SD) age of 40.1 (12) years. The distribution according to Ludwig classification showed type I at 60.9%, type II at 30.4%, and type III at 8.8%. At the beginning of the study, we conducted laboratory tests in patients diagnosed with AGA. The baseline characteristics of study patients are presented in Table 1. Test results of eligible patients showed that ferritin and iron levels were closer to the lower limit of normal. Thyroid-stimulating hormone (TSH) levels were consistent with endocrine gynecological standards in all patients. Analysis of sex hormones showed that the patients did not have abnormally elevated levels of DHT, dehydroepiandrosterone sulfate (DHEA-S), androstenedione, and testosterone, while the sex hormone-binding globulin (SHGB) concentration was at the upper limit of normal.

### 3.2. Evaluation of ACM Treatment Effectiveness

**Figure 2:**
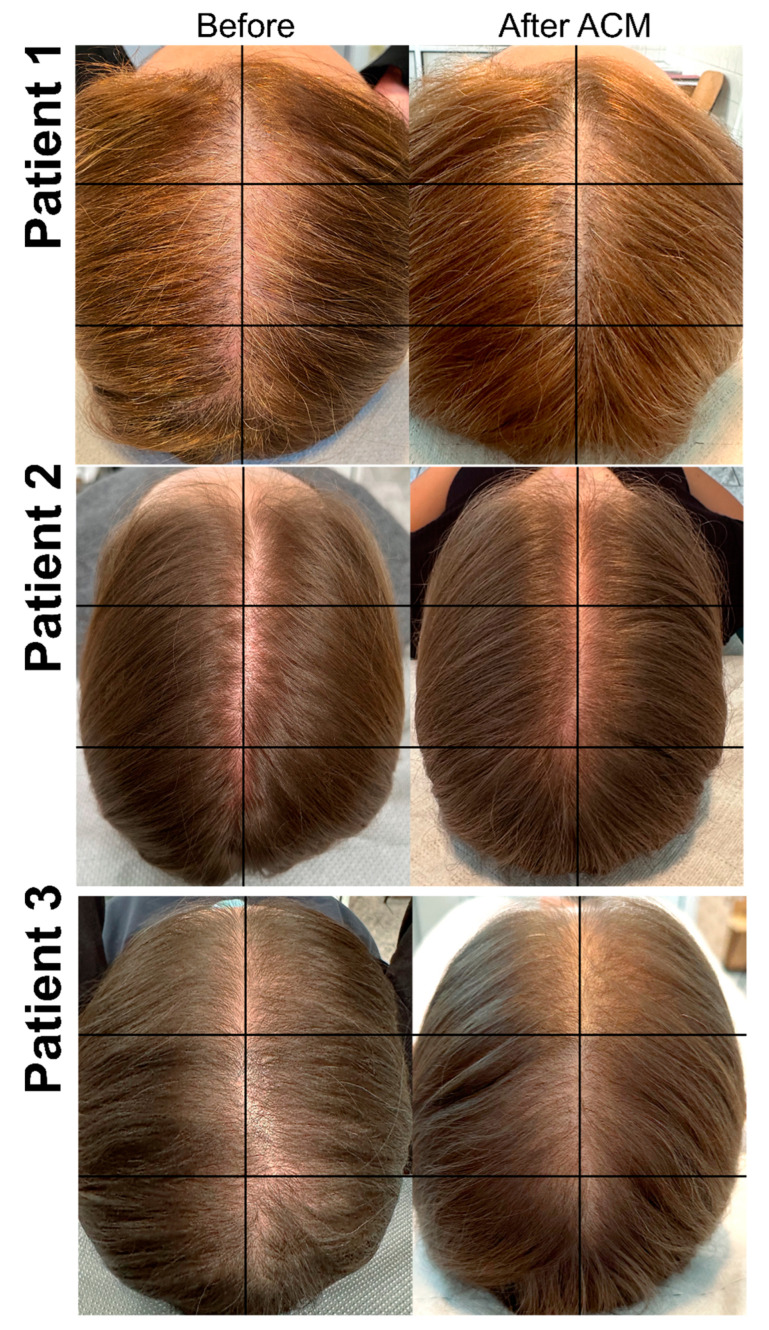
Photographs of sample patients with AGA pre- and post-ACM treatment. **Patient 1.** A 47-year-old female with AGA classified as having stage-2 vertex balding according to the Ludwig scale. Photo before therapy with widening of the central parting. Photo six months after ACM showing improvement in the quantity as well as quality of hair in the central part of scalp. **Patient 2.** A 26-year-old female with AGA classified as having stage-1 vertex balding according to the Ludwig scale. Photo before therapy with noticeable thinning of hair on the top of the head with the normal frontal hairline maintained. Photo after six months after ACM shows an improvement in hair density over the top of the scalp. **Patient 3.** A 39-year-old female with AGA classified as having stage-2 in the Ludwig scale. Photo before therapy with widening of the central parting. Photo six months after ACM showing improvement in the quantity as well as quality of hair in the central part of the scalp.

**Figure 3:**
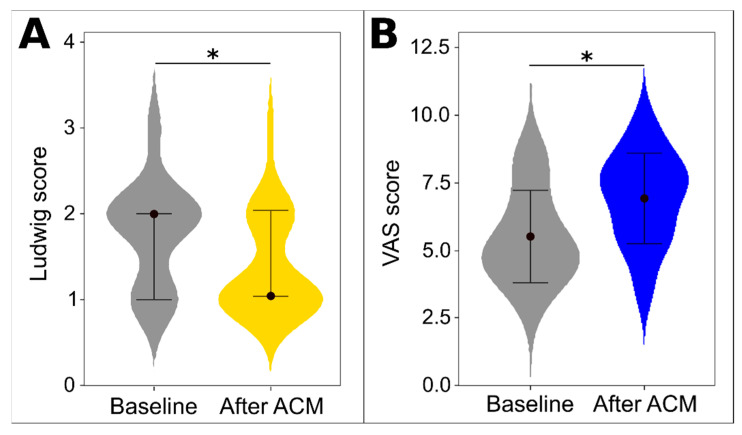
Clinical evaluation of treatment with ACM. Mean Ludwig score (**A**) and mean VAS score from all four specialists (**B**). In plot (**A**), the bars are 1st and 3rd quartile, the dot represents median, and the asterisk (*) indicates a *p*-value of <0.05 from the Wilcoxon signed-rank test. In plot (**B**), the dot represents mean, bars represent standard deviation, and the asterisk (*) indicates a *p*-value of <0.05 from the paired *t*-test.

Patients’ scalp photos (Figure 2) before and six months after therapy with HFSCs were evaluated by four independent specialists. This assessment showed significant improvement based on the VAS scale (Table 2), with an average increase of 1.5 points. After ACM, there was an average improvement of 1 degree on the Ludwig scale. Both Ludwig scale scores and mean VAS scores showed significant differences after treatment as depicted in Figure 3.

### 3.3. Associations between Outcomes and Baseline Characteristics

**Figure 4:**
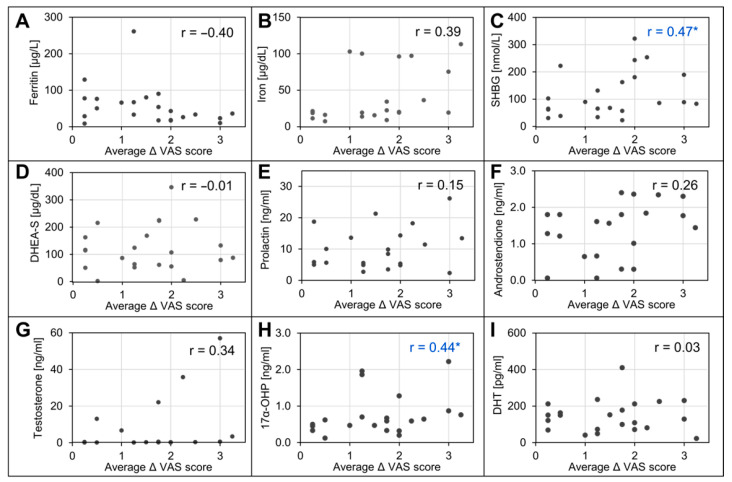
Correlations between changes in VAS score after treatment and baseline clinical characteristics of patients (**A**–**I**). In blue, * *p* < 0.05 is highlighted. Spearman correlation coefficient r for average delta VAS scores (average VAS score after–average VAS score before treatment) for individual independent specialists and mean delta from all specialists. Abbreviations: 17α-OHP, 17α-hydroxyprogesterone DHEA-S, dehydroepiandrosterone sulfate; DHT, dihydrotestosterone; SHGB, sex hormone-binding globulin; TSH, thyroid-stimulating hormone.

The association between the initial blood parameters and clinical outcomes was evaluated based on a calculation of the Spearman correlation coefficients (Figure 4). Upon assessment by half of the specialists, delta VAS scores showed a moderate negative correlation with baseline ferritin concentration and a positive correlation with iron concentration; however, this correlation was not significant when considering the mean delta VAS score. Among sex hormones, the mean better outcomes were associated with higher initial levels of SHGB and 17α-hydroxyprogesterone. Neither testosterone nor DHT showed a significant correlation with VAS scores.

## 4. Discussion

The existing literature on the treatment of female AGA with HFSCs is sparse. In a recent systematic review we conducted, we gathered all available studies pertaining to the use of autologous stem cells of various origins in the treatment of AGA [21]. The use of stem cells in alopecia treatment is a topic of significant interest, yet it remains relatively novel and requires further research. The effectiveness of the ACM treatment with the Regenera^®^ medical device is supported by available evidence, including studies conducted by Gentile et al. [22,36]. In one study, 11 patients showed a 29% ± 5% increase in hair density in the treated area at the 23-week follow-up, compared to less than 1% in the placebo area [36]. In another study, the treated area experienced an average increase of 18.0 hairs compared to baseline after the ACMs were used three times at 45-day intervals [22]. The study by Zari et al., involving 140 patients (113 of whom were female) showed that a single session with Regenera Activa^®^ resulted in an increase in hair density ranging from 4.5 to 7.12 hairs/cm^2^ after six months [23]. The treatment of AGA with HFCSs obtained from autologous micrografts through Regenera^®^ micrografting technology was further evidenced by Ruiz et al. [37]. The study involved 100 patients (both male and female) with measurements of hair density with TrichoScan^®^ two months after the procedure and scalp dermoscopic analysis after four and six months.

Our study further confirmed the effectiveness of the ACM therapy for female patients with AGA. To evaluate the treatment effectiveness, we relied on the VAS scale scores of pre- and post-therapy photos. This approach considers the real cosmetic effect, which is the most significant change as perceived by the patient. Using patient scalp photos as a method for assessing treatment effects might not appear accurate. However, in our opinion, this approach is the most indicative of clinical condition, and it translates directly into the well-being and self-esteem of our patients. The value of photography-based assessment as a measure of visibly significant results was also highlighted in a systematic review and meta-analysis of the effectiveness of AGA treatments [38].

Our secondary aim was to explore the association between the serological characteristics of patients and treatment outcomes. We observed that a higher initial SHGB concentration correlated with a more significant improvement in AGA, as assessed by dermatology specialists. SHGB is a circulating glycoprotein important for transporting sex hormones and regulating their bioavailability [39]. In humans, the role of SHGB is to protect against excess endogenous and exogenic steroids by combining with them [40]. Among female patients, Chen et al. found that low-serum SHGB correlated negatively with AGA severity [41]. Taken together with our observation, this seems to confirm SHGB’s crucial involvement in the presentation of AGA in women.

We did not find any significant deviation in DHT concentrations in our study group, suggesting that AGA may not be dependent on DHT levels. Conflicting evidence exists regarding DHT levels in AGA patients, while Urysiak-Czubatka et al. [42] found no statistically significant difference in DHT levels in patients with AGA compared to healthy controls in a mixed-gender study, suggesting that genetically determined, individualized sensitivity to androgens plays a crucial role in AGA presentation. Zhang et al. reported an increased concentration of DHT in males and a correlation between DHT levels and the curative effect of finasteride [43]. After ACM treatment, we found no association between baseline DHT concentrations and treatment outcomes. This suggests potential sex-based differences that could underlie the degree of therapy effectiveness.

Our study exclusively included women, which did not allow us to stratify the analysis by gender. There are differences between male and female patterns of AGA. The condition is more prevalent in men than in women as it affects about 80% of men and about 50% of women. It typically manifests at an earlier age in men, often beginning in the late teens to early twenties and progressing gradually. Female AGA may onset later, usually after menopause, and its progression can be more variable. In men, the frontal hairline often significantly recedes, forming an “M” shape, while women typically maintain their frontal hairline, and the thinning is more evenly distributed [44,45]. Some hormonal factors can contribute to the picture and pathogenesis of male and female patterns of AGA. In men, AGA is primarily associated with dihydrotestosterone, a byproduct of testosterone, while in women, hormonal fluctuations, including changes in androgens, may contribute to AGA. Nevertheless, the study by Urysiak-Czubatka et al. [42] on the differences in dihydrotestosterone concentration between men and women with AGA yielded inconclusive results, emphasizing the genetically determined sensitivity of the follicles to dihydrotestosterone. This variable sensitivity may be responsible for the diverse reactions to androgens.

Treatment options vary between men and women. Specifically, oral testosterone and its derivatives are not recommended for women, especially those of reproductive age, who may be pregnant or planning to conceive [14,45]. Stem cell therapy and ACM are novel techniques. While some studies have shown promising results, offering substantial benefits to patients, the long-term efficacy is still awaiting confirmation. The study conducted by Zari et al. [23] employed ACMs to treat AGA in both men and women, with a total of 113 female and 27 male participants. Statistically significant improvements were observed in women, particularly in terms of hair density and hair shaft thickness. Specifically, women experienced significant enhancement in hair density in the temporal and occipital scalp regions, while men noted significant improvement in the frontal scalp region. These findings align with the natural course of the disease, demonstrating improvement in the areas most affected in both men and women [44,45]. Considering the pattern of hair loss, Álvarez et al. [29] administered ACM injections in various areas of the scalp in men and women. Their study included 17 participants, of whom eight were women aged between 21 and 58 years. Since this was a descriptive study, the authors concluded that all participants were satisfied with the treatment, noting improvements primarily in thickness and hair loss. Moreover, ACM was well-tolerated with no reported side effects. On the contrary, Gentile et al. [36] exclusively studied a male population. They observed a notable increase in hair density by 29% ± 5% in the treated areas compared to less than 1% in the untreated areas. These findings could suggest that there may be no difference in effectiveness between male and female patterns of AGA.

Over-secretion of adrenal androgens can result from enzyme deficiencies in the biosynthesis of cortisol. Approximately 2% of hyperandrogenic patients present with late adrenal hyperplasia (LOAH) characterized by a lack of 21-hydroxylase [46]. All patients included in our study had levels of 17α-hydroxyprogesterone within the normal range, ruling out hyperandrogenism as the cause of AGA. We noted a positive correlation between 17α-hydroxyprogesterone concentrations and clinical outcomes as assessed by specialists; however, further studies are needed to elucidate the causes of this association.

SC therapies present a novel and promising approach to treating hair loss. Current treatments of AGA often fail to meet the expectations of patients and medical professionals, so there is a substantial demand for new therapies, particularly for individuals with contraindications to standard methods. Our study demonstrated the curative effect of ACM therapy, utilizing the Regenera^®^ procedure.

There were some limitations that should be considered when interpreting the results of our study, one of which was a limited sample size. Furthermore, we did not have access to the technology needed to perform hair evaluation using trichoscopy equipment and hair mapping programs. In the future, we would like to expand our study to include a comparison with one of the FDA-approved treatments. Presently, the FDA endorses the use of two drugs, minoxidil and finasteride, as well as treatment with low-level light/laser therapy devices. The effectiveness of these treatments, compared to placebo, was supported in a recent meta-analysis [38]. Despite the growing interest in alternative therapies, such as the use of growth factors or SCs, there is a scarcity of data on the performance of these strategies relative to the more prevalent forms of treatment. Undoubtedly, the SC-based treatment has some advantages, such as convenience for the patients. The ACM treatment does not face challenges in achieving high compliance, as is the case with oral or topical drugs [47]. Over the course of our study, no adverse effects were reported by the patients. However, there are also some drawbacks. The ACM procedure incurs a significant one-time cost to the patients, and, moreover, the method has not yet been standardized. Nonetheless, the use of the Regenera Activa^®^ device appears to be a promising AGA therapy to explore further, both as a standalone procedure and in conjunction with classical treatments.

## 5. Conclusions

Given the limited treatment options for AGA, there is an increasing demand for alternative therapies that demonstrate acceptable performance levels. This study provides valuable insights into the potential of ACM therapy for AGA and underscores the importance of continued exploration and refinement of the ACM procedure. Our study on the ACM procedure revealed it to be both safe and effective, yielding satisfying results six months after a single treatment session. However, the absence of a standardized procedure highlights the need for further research. Future investigations should aim to gather evidence that enables the development of a cost-effective approach while minimizing treatment burden and costs for patients. Additionally, long-term studies are required to evaluate the sustained effectiveness of the ACM procedure over time.

## Figures and Tables

**Table 1:** Baseline characteristics of study patients.

Variable	Format	Value	Min–Max Value
Age	Mean (SD)	40.1 (12)	25.0–68.0
Vitamin D3 (ng/mL)	Mean (SD)	38.4 (19)	8.00–81.0
Vitamin B12 (pg/mL)	Median (IQR)	260 (181)	123–763
Ferritin (µg/L)	Median (IQR)	43.0 (47)	8.00–261
Iron (µg/dL)	Median (IQR)	20.4 (39)	7.00–113
Folic acid (ng/mL)	Median (IQR)	5.40 (4.5)	3.00–68.0
TSH (µIU/mL)	Median (IQR)	1.41 (1.0)	0.00–3.00
Anti-TPO (IU/mL)	No. over/under norm ^†^	1/22	N/A
Anti-TG (IU/mL)	No. over/under norm ^‡^	1/22	N/A
SHBG (nmol/L)	Median (IQR)	85.4 (112)	22.0–322
ACTH (pg/mL)	Median (IQR)	9.64 (6.9)	1.00–35.0
Cortisol (µg/L)	Median (IQR)	11.4 (10)	3.00–35.0
DHEA-S (μg/dL)	Mean (SD)	131 (91)	1.00–346
Prolactin (ng/mL)	Median (IQR)	5.84 (8.5)	2.00–26.0
Androstenedione (ng/mL)	Mean (SD)	1.43 (1)	0.00–4.00
Testosterone (ng/mL)	Median (IQR)	0.30 (3.36)	0.00–56.0
Hb (g/dL)	Mean (SD)	13.3 (0.9)	11.0–14.0
17α-hydroxyprogesterone (ng/mL)	Median (IQR)	0.59 (0.4)	0.00–2.00
DHT (pg/mL)	Median (IQR)	134 (118)	22.0–409
ANA	No. positive/negative	7/16	N/A
Ludwig scale score	1/2/3	7/14/2	N/A

^†^ norm is up to 35 IU/mL; ^‡^ norm is up to 40 IU/mL. ACTH, adrenocorticotropin; ANA; anti-nuclear antibodies, Anti-TG, antithyroglobulin antibodies; Anti-TPO, anti-thyroid peroxidase antibodies; DHEA-S, dehydroepiandrosterone sulfate; DHT, dihydrotestosterone; Hb, hemoglobin; N/A, not applicable; SHGB, sex hormone-binding globulin; TSH, thyroid-stimulating hormone.

**Table 2:** VAS scoring results.

Variable	Format	After	*p* Value ^†^	Value
Specialist 1	5 (2)	7 (2)	<0.001	40.1 (12)
Specialist 2	6 (2.5)	8 (2.5)	<0.001	38.4 (19)
Specialist 3	5 (2.5)	7 (2)	<0.001	260 (181)
Specialist 4	5 (2)	7 (2)	<0.001	43.0 (47)

^†^ paired *t*-test; values are medians (IQR).

## Data Availability

The data that support the findings of this study are available on request from the corresponding author.
